# New York

**Published:** 1896-12

**Authors:** 


*New York.* In the northern part of New York City, out of a herd of 139 cows, 27 were found to be tuberculous when tested. Most of these animals were from one herd, which had been supplying with milk the New York Juvenile Asylum and the New York Institution for the Instruction of the Deaf and Dumb. No more serious state of affairs could exist than to learn that public institutions of a charitable character were being supplied with milk from such dangerous sources, and it must be evident by this time that the first work for every State in the Union to do is to investigate this source of food-supply for the inmates of all public institutions. It is reported that the health department of New York City will undertake at once the prevention of the sale of tuberculous milk brought into the city from any source whatever. This means, of course, an examination of all the herds supplying the city with milk.

